# Paediatric Appendicectomy in a UK District General Hospital: Retrospective Analysis of Laparoscopic Versus Open Approaches Performed by Non-Paediatric Specialist General Surgeons

**DOI:** 10.7759/cureus.97717

**Published:** 2025-11-24

**Authors:** Sara Azeem, Zohaib Jamal, Leena S Harishkumar, Anza T Khan, Khizra Zafar, Imran Alam

**Affiliations:** 1 Department of Surgery, Wrightington, Wigan and Leigh NHS Foundation Trust, Wigan, GBR; 2 Department of Surgery, Railway General Hospital, Rawalpindi, PAK; 3 Consultant General and Benign Upper Gastrointestinal, Wrightington, Wigan and Leigh NHS Foundation Trust, Wigan, GBR

**Keywords:** laparoscopic pediatric surgery, laparoscopic versus open surgery, open appendicectomy, pediatric appendicectomy, surgical outcomes

## Abstract

Background

Acute appendicitis remains the most frequent paediatric surgical emergency worldwide. Although laparoscopic appendicectomy has progressively supplanted the traditional open approach in specialist paediatric centres, evidence regarding its outcomes in non-specialist District General Hospitals remains limited. In such settings, appendicectomies in children are frequently performed by general surgeons, introducing potential variability in outcomes related to surgeon experience and institutional resources.

Methods

A retrospective cohort study was conducted at Wrightington, Wigan and Leigh NHS Foundation Trust, United Kingdom. All patients aged ≤16 years who underwent emergency appendicectomy for suspected acute appendicitis between 1st January 2016 and 31st August 2025 were included. The primary outcome was the immediate postoperative complications within the same admission and a 30-day postoperative admission rate, including surgical site infection, intra-abdominal abscess, and postoperative ileus. Secondary outcomes included operative duration, length of hospital stay, and conversion rate. Statistical analysis was performed using SPSS v28.0. A p-value <0.05 was considered statistically significant.

Results

A total of 254 paediatric patients underwent appendicectomy for suspected acute appendicitis between January 2016 and August 2025. Following exclusion of incomplete records, 237 patients were included in the final analysis, comprising 199 (84.0%) open appendicectomies (OA), 36 (15.2%) laparoscopic appendicectomies (LA), and 2 (0.8%) laparoscopic procedures converted to open. The mean patient age was 10.9 ± 2.7 years, with a male predominance (63.3%). The mean American Society of Anesthesiologists (ASA) grade was 1.21 in the OA group and 1.25 in the LA group. Preoperative imaging was performed in 101 (42.6%) cases, most commonly ultrasound, with comparable diagnostic accuracy between groups (*p = 0.99*). The mean operative duration was 70.0 minutes, with median durations of 63.8, 97.2, and 195.0 minutes for open, laparoscopic, and converted cases, respectively. Complicated appendicitis occurred in 58 (28.9%) OA and 10 (27.8%) LA cases (*p = 0.89*). Drain placement was required in 26 (12.9%) OA and 7 (19.4%) LA cases (*p = 0.31*). The overall postoperative complication rate was 23 (9.7%), most frequently due to postoperative ileus (8 (3.4%)) and intra-abdominal abscess (8 (3.4%)), with no significant difference between surgical approaches. The 30-day readmission rate was 26 (10.9%), predominantly for intra-abdominal collections (10 (4.2%)) and postoperative pain (7 (3.0%)). The negative appendicectomy rate was 13 (5.5%), lower than national averages (~10-13%). The majority of procedures (174 (73.4%)) were performed by registrars under consultant supervision, reflecting the reproducibility of both approaches in a training environment. No intraoperative access-related complications or major adverse events were recorded.

Conclusion

Laparoscopic appendicectomy performed by general surgeons in a District General Hospital is safe and effective for paediatric patients, demonstrating outcomes comparable to open surgery with low complication and negative appendicectomy rates. The high proportion of registrar-performed procedures highlights its reproducibility in a training environment. These findings support the routine use of laparoscopic appendicectomy in non-specialist centres with appropriate training and resources.

## Introduction

Acute appendicitis is the most common paediatric surgical emergency worldwide, with appendicectomy remaining the definitive treatment [1]. Over the past three decades, surgical practice has undergone a profound transformation: the classical open appendicectomy via a right iliac fossa incision has increasingly been supplanted by minimally invasive laparoscopic appendicectomy, reflecting a paradigm shift towards enhanced recovery, reduced wound morbidity, and shorter hospital stay [1,2]. This transition has been primarily driven by the proposed advantages of the laparoscopic approach, including reduced postoperative pain, superior cosmetic outcomes, and accelerated resumption of normal daily activities [2].

Within paediatric surgical practice, the implementation of minimally invasive modalities has been meticulously appraised, with careful consideration of the distinctive physiological and anatomical attributes of children. A substantial body of high-quality evidence, including large-scale studies and meta-analyses, has consistently demonstrated that LA confers significant clinical benefits over the traditional OA, notably in the form of reduced surgical site infection (SSI) rates and abbreviated hospital stays [1,3]. These advantages persist even in the context of complicated appendicitis, such as perforated or gangrenous presentations, where LA has been validated as a safe and efficacious alternative to OA [4]. The minimally invasive approach facilitates comprehensive inspection of the peritoneal cavity, providing a particular advantage in cases with diagnostic uncertainty or atypical appendix positioning, while the reduced wound burden inherently diminishes the risk of postoperative infectious complications [2,4].

In paediatric surgical care, the utilisation of minimally invasive techniques has been subject to meticulous scrutiny, particularly in settings where children are managed within non-specialist institutions. In the United Kingdom, a substantial number of paediatric appendicectomies are performed in District General Hospitals (DGHs), often under the care of consultant general surgeons whose principal training and routine practice focus on adult patients [5]. Within this framework, several distinctive variables emerge that may modulate operative and postoperative outcomes. These include the individual surgeon’s caseload and experiential familiarity with paediatric laparoscopy, the accessibility of appropriately sized paediatric instrumentation and ancillary equipment, and the configuration of peri‑operative support, namely anaesthetic teams and nursing staff whose exposure to emergency paediatric surgery may be limited relative to specialist paediatric centres [6].

In the existing literature, the preponderance of evidence concerning the comparative efficacy of laparoscopic versus open appendicectomy in children derives from tertiary paediatric hospitals and large academic centres, thereby constraining the generalisability of those findings to non-specialist institutions [5]. The extent to which the advantages of LA, as delineated within specialist paediatric centres, are generalisable to the DGH setting, where consultant general surgeons with predominantly adult-oriented practice are the principal operators, remains indeterminate [5,6]. Concerns persist regarding the potential for prolonged operative duration, increased conversion rates, and divergent complication profiles when laparoscopic techniques are applied by adult general surgeons to smaller paediatric patients [5,6,7]. Accordingly, a focused evaluation of outcomes within the DGH setting is imperative to substantiate current surgical practices, guide clinical governance frameworks, and ensure the maintenance of equitable standards of care irrespective of institutional context.

This study aims to bridge the existing knowledge gap by undertaking a retrospective comparative analysis of laparoscopic versus open appendicectomy in patients under 16 years of age treated at a single United Kingdom DGH. Through evaluation of key clinical metrics, including but not limited to postoperative complication rates, length of hospital stay, and operative duration, the study seeks to ascertain whether the established benefits of laparoscopic surgery, as demonstrated in specialist paediatric centres, are reproducible within a non-specialist DGH environment. The findings are intended to contribute evidence-based insight into the safety, efficacy, and applicability of minimally invasive appendicectomy in general surgical practice, thereby informing both clinical governance and service delivery models across varying tiers of healthcare provision.

## Materials and methods

Study design and setting

This retrospective cohort study was undertaken at Wrightington, Wigan and Leigh NHS Foundation Trust, a single NHS DGH in the United Kingdom. The retrospective design facilitated a comprehensive assessment of real-world clinical outcomes associated with laparoscopic versus open appendicectomy in paediatric patients managed by adult general surgeons, through systematic analysis of routinely collected clinical data. The study context mirrors the operational framework of many non-specialist centres across the UK, where paediatric surgical emergencies are predominantly managed by general surgical teams. All data were anonymised and analysed following approval from the institution’s Clinical Audit and Governance Department, which granted exemption from individual patient consent due to the retrospective and non-interventional nature of the investigation. The study was conducted in accordance with the ethical principles outlined in the Declaration of Helsinki.

Patient population and selection criteria

The study included all patients aged ≤16 years who underwent emergency appendicectomy for suspected acute appendicitis between 1st January 2016 and 31st August 2025. This age threshold reflects the conventional definition of paediatric care in the UK DGH setting. Eligible patients were identified via the hospital’s electronic theatre management and patient record systems using standardised procedural codes.

Inclusion Criteria

1. Patients aged 16 years or younger at the time of surgery; 2. emergency presentation with clinical or radiological suspicion of acute appendicitis; 3. underwent either a laparoscopic or an open appendicectomy; 4. histopathological analysis of appendicitis on the resected specimen.

Exclusion Criteria

1. Patients over the age of 16; 2. Patients who underwent an elective or interval appendicectomy; 3. Incidental appendicectomies performed during other abdominal procedures; 4. Patients with incomplete or missing critical data points in their medical records that would preclude meaningful analysis of primary or secondary outcomes; 5. Patients transferred to a tertiary paediatric surgical centre intra-operatively or immediately postoperatively before primary outcomes could be assessed.

In our institution, assignment to laparoscopic versus open appendicectomy was not determined by predefined selection criteria. Instead, the operative approach was influenced by several pragmatic factors including: (1) surgeon preference and familiarity with paediatric laparoscopy; (2) availability of appropriately sized laparoscopic instruments, particularly earlier in the study period; (3) patient age and body habitus-where younger and smaller children were more commonly managed via an open approach; and (4) the clinical severity at presentation, with some cases of advanced peritonitis or haemodynamic instability proceeding directly to open surgery at the discretion of the operating consultant. These factors reflect real-world operative decision-making in non-specialist DGH settings and account for the higher proportion of open procedures

Data collection and variables

Data were retrospectively extracted from the hospital’s electronic patient record (EPR) system using a standardised proforma, incorporating clinical notes, operative records, anaesthetic charts, pathology reports, and discharge summaries. To ensure data reliability, two independent investigators performed the data collection, with any discrepancies resolved by a third senior reviewer. All data were de-identified and securely stored on a password-protected database. Collected variables included patient demographics (age, sex, and body mass index), preoperative parameters (admission date, symptom duration, clinical findings such as localised or generalised peritonitis, inflammatory markers including white blood cell count and C-reactive protein, imaging findings from ultrasound or CT, and ASA grade), intraoperative details (surgical approach, operative duration from skin incision to closure, conversion to open surgery, intraoperative findings categorised as simple or complicated appendicitis, and any intraoperative complications such as haemorrhage or visceral injury), and postoperative outcomes (histopathology results, length of hospital stay, immediate postoperative complications within the same admission, and 30-day readmission rates).

Statistical analysis

All statistical analyses were performed using SPSS software (Version 28.0, IBM Corp., Armonk, NY). Data were analysed using standard descriptive and comparative statistical methods. Categorical variables were summarised as frequencies and percentages, while continuous variables were reported as means with standard deviations or medians with ranges, depending on distribution. Comparisons between the laparoscopic and open appendicectomy groups were made using the chi-square test or Fisher’s exact test for categorical variables, and the independent t-test or Mann-Whitney U test for continuous variables. Data are presented as mean ± standard deviation (SD) for continuous variables and as number (percentage) for categorical variables. A p-value of <0.05 was considered statistically significant.

## Results

**Figure 1:**
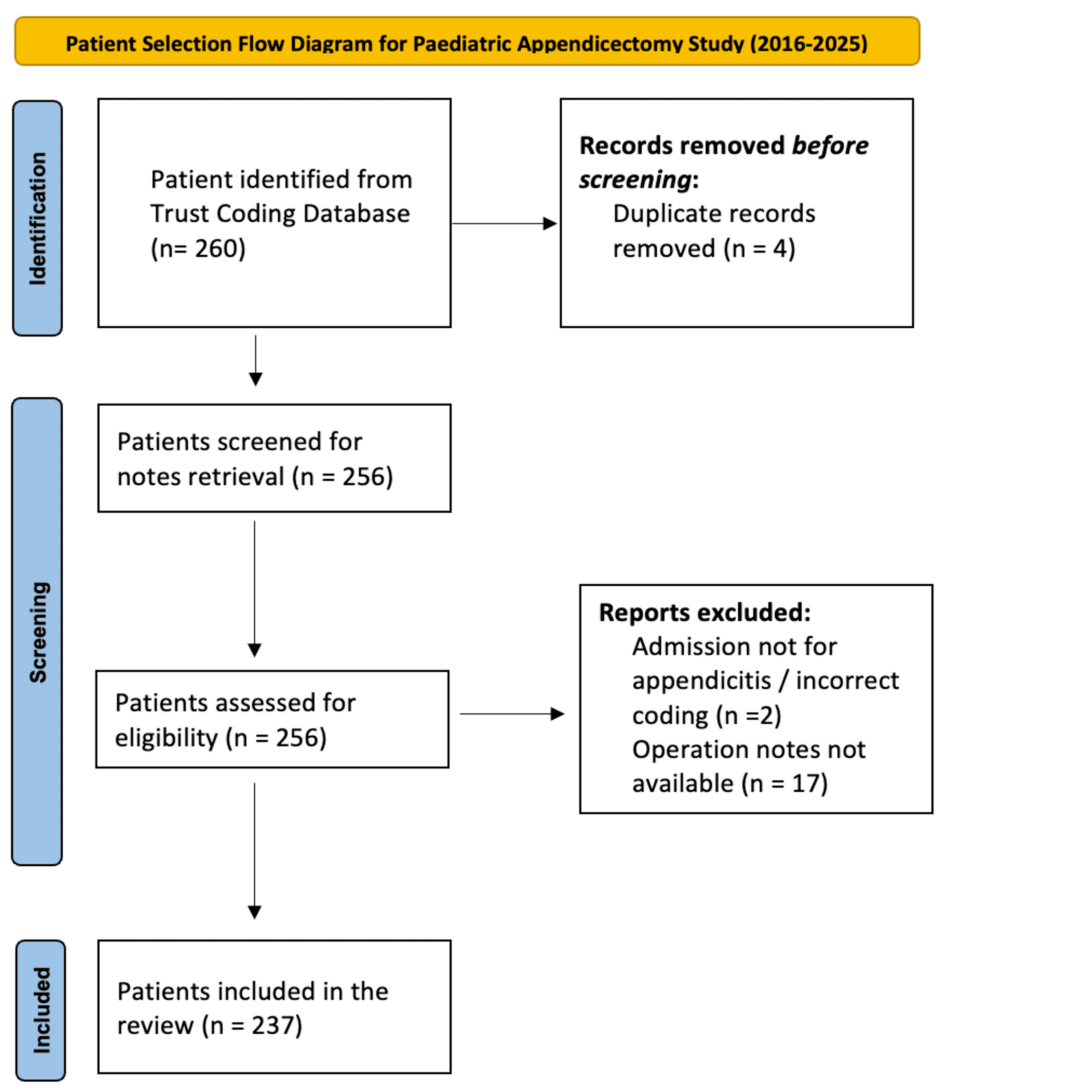
Study Flow Diagram for Paediatric Appendicectomy – Case Identification to Inclusion

Between 1st January 2016 and 31st August 2025, a total of 256 paediatric patients underwent appendicectomy for suspected acute appendicitis at WWL NHS Foundation Trust. Following the application of exclusion criteria, 237 patients with complete clinical data were included in the final analysis (Figure 1). Among these, 199 patients (84.0%) underwent an OA, 36 patients (15.2%) underwent an LA, and 2 patients (0.8%) underwent a laparoscopic procedure converted to open. The higher proportion of OAs (84%) is reflective of local practice patterns, surgeon expertise distribution, and limited paediatric laparoscopic instrument availability in the earlier years of the study period.

Patient demographics

The demographic and baseline clinical characteristics of the two cohorts are summarised in Table 1. The mean age of the overall study population was 10.9 ± 2.7 years. Patients in the LA group were older, with a mean age of 13.4 ± 2.2 years, compared to 10.4 ± 2.6 years in the OA group. Across the entire cohort, 150 (63.3%) patients were male and 87 (36.7%) were female, resulting in an overall male-to-female ratio of approximately 1.7:1. The mean ASA grade was 1.21 in the OA group and 1.25 in the LA group, indicating that both cohorts were comparable in terms of baseline physical status. The majority of patients in both groups were classified as ASA I-II. A comparable proportion of patients in each group presented with complicated appendicitis, defined as perforation, gangrene, or abscess formation (58 (28.9%) in the OA group vs. 10 (27.8%) in the LA group). These findings suggest that the two groups were generally well-matched in terms of baseline health and disease severity.

**Table 1:** Demographic and Baseline Characteristics of Patients Undergoing Paediatric Appendicectomy Data are presented as median (range) or number (percentage). Statistical comparisons were performed using the independent t-test for continuous variables and the chi-square or Fisher’s exact test for categorical variables. Statistical significance is defined as p < 0.05. ASA: American Society of Anesthesiologists; LA: laparoscopic appendicectomy; OA: open appendicectomy

Variable	OA Group (n = 201)	LA Group (=36)
Median age (years)	10 (range 5–15)	14 (range 6–15)
Male	132 (65.6%)	18 (50.0%)
Female	69 (34.3%)	18 (50.0%)
ASA I	159 (79.1%)	28 (77.77%)
ASA II	40 (19.9%)	9 (25%)
ASA III	0 (0.0%)	1 (2.77%)
Median weight (kg)	63.0 (range 32.1–94.4)	38.0 (range 18.7–85.9)

Preoperative imaging

Preoperative imaging was more frequently performed in the laparoscopic group (21 (58.3%)) than in the open group (80 (39.8%)), although this difference was not statistically significant (p = 0.06). Ultrasound was the predominant imaging modality, and the diagnostic accuracy for appendicitis was comparable between the two cohorts (65.0% in OA vs. 61.9% in LA; p = 0.99). Table 2 presents detailed preoperative imaging characteristics.

**Table 2:** Preoperative Imaging Characteristics of Patients Undergoing Paediatric Appendicectomy Statistical comparisons were performed using the chi-square or Fisher’s exact test for categorical variables. Statistical significance is defined as p < 0.05. US: ultrasound; CTAP: computed tomography of the abdomen and pelvis; MRI: magnetic resonance imaging; LA: laparoscopic appendicectomy; OA: open appendicectomy

Variable	OA group (n = 201)	LA group (n = 36)	p-value (χ²)
Preoperative imaging performed	80 (39.8%)	21 (58.33%)	χ² = 3.53, p = 0.06
Imaging diagnostic of appendicitis	52 / 80 (65.0%)	13 / 21 (61.9%)	χ² = 0.01, p = 0.99
Imaging modality			
Ultrasound (US)	44 (55.0%)	9 (42.9%)	—
CT abdomen/pelvis (CTAP)	29 (36.2%)	8 (38.1%)	—
MRI abdomen	7 (8.8%)	4 (19.0%)	—

Intraoperative outcomes

**Figure 2:**
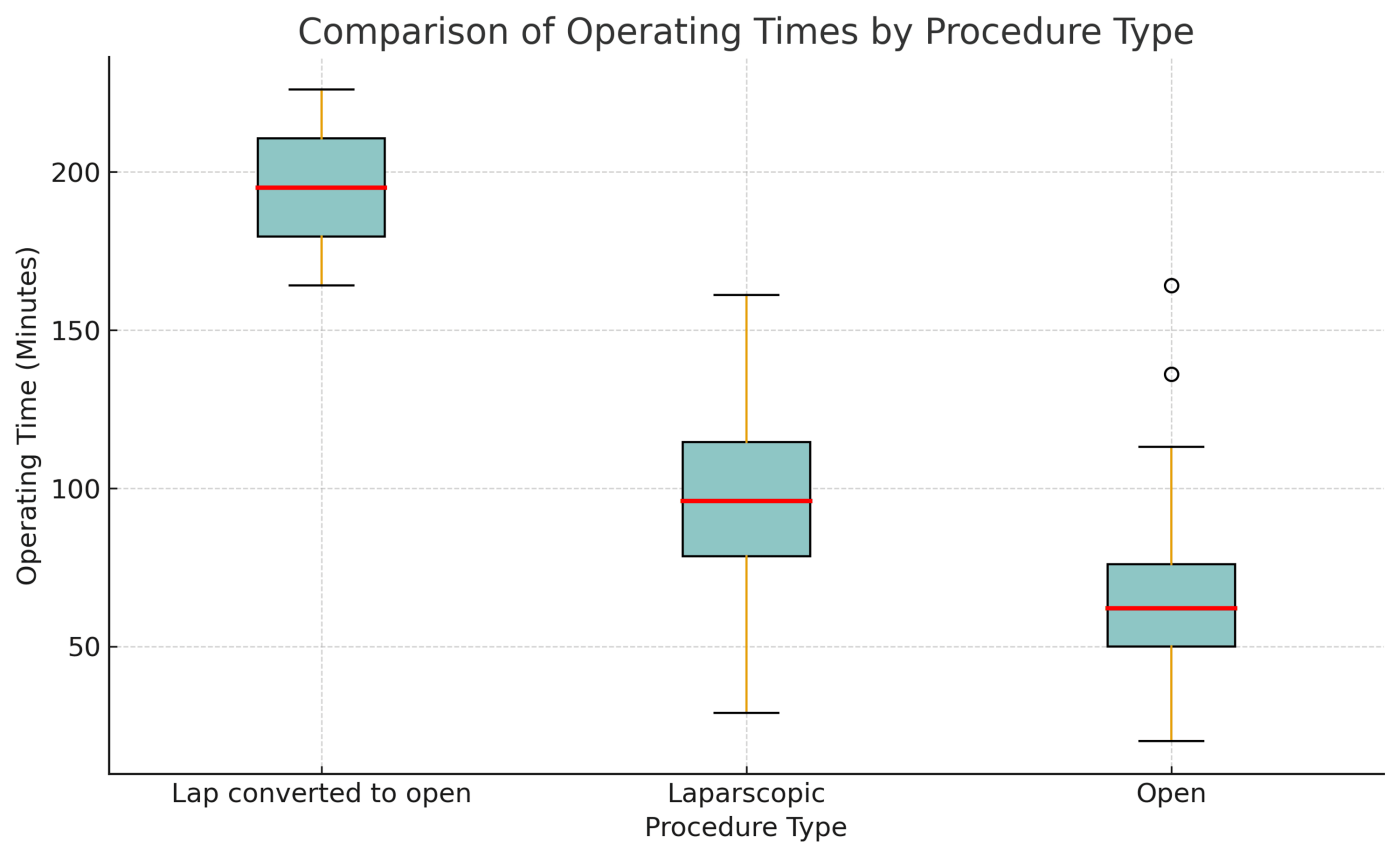
Comparison of Operative Duration by Surgical Approach in Appendicectomy Procedures Operative durations represented as median (interquartile range).

The mean operative duration for the cohort was 70.0 minutes. Figure 2 presents a comparative box plot of operative durations for open, laparoscopic, and laparoscopic-converted-to-OAs. The median operative time was shortest for open procedures (63.8 minutes), longer for laparoscopic cases (97.2 minutes), and substantially prolonged in converted cases (195.0 minutes). The interquartile range was broader for laparoscopic procedures, indicating greater variability in operative duration, whereas converted cases exhibited consistently extended times. This distribution reflects the anticipated escalation in operative time associated with increasing procedural complexity.

Rates of complicated appendicitis and drain placement were similar between the two groups, with no statistically significant differences observed. Among complicated cases, perforated appendicitis was the predominant subtype, followed by appendicular abscess formation. The majority of operations were undertaken by registrars, with consultants and senior house officers performing a smaller proportion. No intraoperative access- or port-related complications occurred, and intraoperative blood loss was minimal, with no patient requiring blood transfusion. Table 3 summarises the intraoperative characteristics and operating surgeon grades stratified by procedure type.

**Table 3:** Intraoperative Characteristics and Operating Surgeon Grade by Procedure Type Data are presented as a percentage (number). Statistical comparisons were performed using the Mann–Whitney U test for continuous variables and the chi-square or Fisher’s exact test for categorical variables. Statistical significance is defined as p < 0.05. LA: laparoscopic appendicectomy; OA: open appendicectomy; SHO: senior house officer

Variable	OA Group (n = 201)	LA Group (n = 36)	p-value (U or χ²)
Complicated appendicitis (%)	28.8% (n = 58)	27.7% (n = 10)	χ² = 0.02, p = 0.89
Drain placement (%)	12.9% (n = 26)	19.4% (n = 7)	χ² = 1.03, p = 0.31
Primary operating surgeon			
Consultant (%)	25.3% (n = 51)	27.7% (n = 10)	—
Registrar (%)	73.6% (n = 148)	72.2% (n = 26)	—
SHO (%)	0.99% (n = 2)	0 (n = 0)	—

Postoperative outcomes

The mean postoperative length of stay (LOS) across the cohort was 1.94 ± 1.54 days, with no statistically significant difference observed between the open and laparoscopic groups. Postoperative complications within the same admission occurred in 23 patients (9.7%) overall. The most frequent complications were postoperative ileus (8 (3.4%)) and intra-abdominal abscess formation (8 (3.4%)), followed by SSI (4 (1.7%)). Less common postoperative complications included Clostridium difficile infection, adhesive small bowel obstruction, and wound dehiscence, which together accounted for 3 (1.3%) of cases. The patient with bowel obstruction was diagnosed with a closed-loop obstruction secondary to intra-abdominal adhesions and was subsequently transferred to a tertiary care centre for further surgical management. The patient with C. difficile infection was managed conservatively and discharged home on a 10-day course of oral vancomycin.

The overall 30-day readmission rate was 10.9% (n = 26), most commonly for intra-abdominal collections or fluid-related complications (10 (4.2%)), postoperative pain (7 (3.0%)), or wound issues (3 (1.3%)). A small proportion of readmissions were related to non-surgical causes, including constipation, dermatological reactions, and unrelated outpatient reviews. No postoperative mortality was recorded. No statistically significant differences were found between OA and LA groups for postoperative complications or readmission causes (all p > 0.05). The OA group showed slightly higher rates of complications, especially intra-abdominal abscesses and wound-related issues, but these differences were not significant. The readmission pattern was broadly similar between groups, with fluid collections and postoperative pain being the most frequent causes.

Table 4 presents postoperative morbidity, length of hospital stay, and readmission patterns comparing open appendicectomy (OA) and laparoscopic appendicectomy (LA).

**Table 4:** Postoperative Morbidity, Length of Stay, and Readmission Patterns Following OA versus LA Data are presented as mean ± standard deviation (SD) or percentage (number), unless otherwise specified. Statistical comparisons were performed using the independent t-test for continuous variables and the chi-square or Fisher’s exact test (t and χ² values reported) for categorical variables. Statistical significance is defined as p < 0.05.

Variable	OA group (n = 201)	LA group (n = 36)	Test statistic (t, χ²)	p-value
Mean postoperative LOS (days)	1.96 ± 1.48	1.84 ± 1.63	t = 0.81	0.42
Overall complication rate (%)	20 (9.95%)	3 (8.33%)	χ² = 1.70	0.19
Type of complication				
– Ileus	6 (2.98%)	2 (5.55%)	χ² = 0.88	0.35
– Intra-abdominal abscess	7 (3.48%)	1 (2.77%)	χ² = 0.00	1.00
– Surgical site infection	4 (1.99%)	0 (0.0%)	χ² = 0.00	1.00
– Other (stump appendicitis, Clostridioides difficile, obstruction, wound reopening)	3 (1.49%)	0 (0.0%)	χ² = 0.00	1.00
30-day readmission (%)	21 (10.44%)	5 (13.8%)	χ² = 0.34	0.56
Common readmission causes				
– Fluid/collection-related issues	7 (3.48%)	3 (8.33%)	χ² = 1.80	0.18
– Postoperative pain	6 (2.98%)	1 (2.77%)	χ² = 0.00	1.00
– Abdominal pain (non-surgical)	2 (0.99%)	0 (0.0%)	χ² = 0.00	1.00
– Wound infection/drainage	2 (0.99%)	1 (2.77%)	χ² = 0.73	0.39
– Other minor causes	4 (1.99%)	1 (2.77%)	χ² = 0.33	0.56

Histopathological findings

The histopathological negative appendicectomy rate across the cohort was low, recorded in 13 patients (5.5%). Within individual subgroups, negative appendicectomy was observed in 11 (5.5%) cases of the open appendicectomy (OA) group and in 2 (5.6%) cases of the laparoscopic appendicectomy (LA) group. This difference was not statistically significant (p = 0.45). Other histopathological findings were rare. A single case (1 (0.5%)) of Enterobius vermicularis infestation was identified in the OA group, with no corresponding cases in the LA cohort (p = 1.00). No instances of appendiceal neoplasm or malignancy were reported in either group. These findings indicate that both surgical approaches were associated with a low rate of negative appendicectomies and an absence of unexpected pathological diagnoses, suggesting appropriate case selection and diagnostic accuracy across the study cohort.

## Discussion

This study presents a comparative evaluation of LA and OA in paediatric patients under the age of 16, performed by non-paediatric specialist adult general surgeons within a DGH setting. The findings substantiate that LA constitutes a safe and technically feasible intervention in this context, demonstrating outcomes comparable to those of the open approach with respect to postoperative morbidity, readmission frequency, and histopathological diagnostic accuracy.

The overall postoperative complication rate observed in the present study was 9.7%, aligning closely with rates reported in previously published literature from both specialist and non-specialist centres [5,8]. The most frequently encountered complications were postoperative ileus and intra-abdominal abscess, with no statistically significant difference identified between the laparoscopic and open cohorts. These findings are concordant with those of the national prospective observational study by Sogbodjor et al., which analysed 2,799 paediatric patients across 80 UK hospitals who underwent surgery for suspected appendicitis and reported an overall postoperative complication rate of 7% [9]. Similarly, in a single-centre study by Hannan et al., the postoperative complication rate was 0% in the laparoscopic appendicectomy (LA) group compared with 11.8% in the open appendicectomy (OA) group (p = 0.01), comparable findings were demonstrated in the present study, further reinforcing the safety and efficacy of the laparoscopic approach in paediatric patients operated on by general surgeons [10]. Collectively, these results corroborate prior meta-analyses demonstrating the non-inferiority of laparoscopic appendicectomy in paediatric populations, even when performed by general surgeons [7, 11]. The absence of access-related injuries or major intraoperative complications within the current series further substantiates the safety, technical feasibility, and reproducibility of the laparoscopic approach when undertaken in a DGH environment equipped with appropriate surgical expertise and institutional infrastructure.

The 30-day readmission rate observed in the present study was 10.9%, marginally exceeding the rates reported in national and multicentre datasets. Giuliani et al. conducted a comprehensive population-based analysis of paediatric emergency appendicectomies across England, reporting readmission rates of 6.6% in DGHs and 6.0% in specialist paediatric centres, respectively [5]. Similarly, Rice-Townsend et al. documented a 30-day readmission rate of 8.7% among children undergoing appendicectomy across freestanding paediatric hospitals in the United States, while a UK-based audit by Sinha et al. identified an overall readmission rate of 10.5% across paediatric surgical procedures [12,13]. The comparable distribution of readmission causes between groups in the present study, predominantly fluid collections and postoperative pain, suggests that these readmissions were not directly attributable to the chosen surgical approach. Rather, they likely reflect the expected spectrum of postoperative variation inherent to emergency paediatric surgical populations, underscoring the multifactorial nature of readmission following appendicectomy. It is important to acknowledge that the predominance of open cases does not reflect intentional selection but rather systemic and logistical factors typical of DGH environments, including variable exposure to paediatric laparoscopy among adult general surgeons.

The histopathological negative appendicectomy rate in the present study was 5.5%, which is lower than national figures reported across large-scale UK datasets. Analyses of the Hospital Episode Statistics HES by Giuliani et al. demonstrated negative appendicectomy rates ranging between 10% and 13% in children, with a 28% lower risk observed in specialist paediatric centres compared to DGHs [5]. These findings are consistent with Getting It Right First Time (GIRFT) national benchmarking guidance, which recommends a target rate of approximately 5% as indicative of appropriate diagnostic and surgical selectivity [14]. Moreover, contemporary UK single-centre series, such as that by Hannan et al., have reported rates at the lower end of the national spectrum (4-5%) [10]. When compared to global data, recent meta-analyses, including that by Henriksen et al. (2023), report a pooled negative appendicectomy rate of approximately 13% (95% CI 12-14%), consistent with the international benchmark [15]. Collectively, these data suggest that the diagnostic and operative thresholds employed in this cohort were appropriately selective, achieving a low non-therapeutic appendicectomy rate without compromising clinical safety.

Operative duration and intraoperative metrics, including conversion rates and drain placement, were similar between groups. The finding that over 70% of operations were performed by registrars under consultant supervision demonstrates the reproducibility of both surgical approaches in a training environment. This supports the growing evidence that laparoscopic paediatric appendicectomy can be safely integrated into general surgical practice without compromising patient outcomes [4, 16].

Collectively, these findings contribute to the expanding body of evidence supporting the capacity of DGHs to deliver safe and effective paediatric emergency surgical care. The comparable clinical outcomes observed between laparoscopic and open appendicectomy indicate that, with appropriate surgical training, access to suitable instrumentation, and robust institutional governance, laparoscopic appendicectomy may be routinely implemented as the preferred operative approach for paediatric appendicitis, even in non-specialist settings.

Strengths and limitations

A principal strength of the present study lies in its reflection of real-world surgical practice within a non-tertiary, DGH context, thereby providing a pragmatic assessment of outcomes achievable by general surgeons performing emergency paediatric procedures within the NHS framework. The inclusion of a large, consecutive cohort spanning nearly a decade strengthens the external validity and temporal robustness of the findings, offering a representative overview of evolving surgical trends and outcomes over time.

Nonetheless, certain methodological limitations warrant consideration. The retrospective design inherently constrains the ability to control for potential confounding variables, including surgeon experience, intraoperative judgement, and variability in case complexity. Furthermore, the single-centre nature of the dataset may restrict the generalisability of the results to other institutional settings. Incomplete documentation of specific perioperative parameters-such as postoperative pain scores, intraoperative findings, and antibiotic duration-further limits the granularity of comparative analysis between surgical approaches. The unequal allocation between groups - where a significantly greater number of procedures were performed via the open approach - was driven by surgeon expertise, patient age, and equipment availability. This non-randomised distribution reflects real-world DGH practice but introduces potential selection bias and limits the comparative statistical power.

Clinical implications and future directions

The findings of this study carry significant implications for contemporary clinical practice. They provide evidence that laparoscopic appendicectomy can be safely and effectively performed in paediatric patients by adult general surgeons within DGH settings, contingent upon appropriate clinical governance, access to suitable instrumentation, and structured operative training. Broader integration of laparoscopic capability in general hospitals has the potential to reduce unnecessary interhospital transfers, enhance service efficiency, and improve continuity of care for paediatric emergency surgical patients.

Future research should prioritise multicentre, prospective studies to corroborate these findings across diverse institutional contexts and to incorporate patient-reported outcome measures such as postoperative pain, recovery time, and quality of life. Moreover, formal cost-effectiveness evaluations and workforce-based analyses could further delineate the economic and operational benefits of routine laparoscopic implementation within DGH environments, thereby informing national service planning and training policy.

## Conclusions

This study provides compelling evidence that LA, when performed by non-paediatric specialist - adult general surgeons within a DGH setting, is a safe and effective operative approach for paediatric patients under 16 years of age. Clinical outcomes, including postoperative complication rates, readmission frequencies, and histopathological accuracy, were comparable to those reported by specialist paediatric surgical centres, thereby reinforcing the technical feasibility and clinical safety of LA in non-specialist environments. The overall postoperative complication rate (9.7%) and negative appendicectomy rate (5.5%) were consistent with established national benchmarks and international data, reflecting the diagnostic precision and operative standards achievable in this context. Furthermore, the predominance of procedures undertaken by registrars under consultant supervision underscores the reproducibility of both laparoscopic and open approaches within a structured surgical training framework. These findings highlight the important role of DGHs in providing high-quality paediatric emergency surgical care, demonstrating that, with adequate training, institutional governance, and resource allocation, laparoscopic appendicectomy can be safely integrated as the preferred standard of care. Future multicentre prospective studies are warranted to corroborate these results, incorporate patient-reported outcome measures, and evaluate the cost-effectiveness and service-level implications of expanding paediatric laparoscopic capability across general surgical networks.
